# Pneumoperitoneum, pneumatosis intestinalis and portal venous gas: Rare gastrostomy complications case report

**DOI:** 10.1016/j.ijscr.2019.04.018

**Published:** 2019-04-28

**Authors:** Carlos Jose Perez Rivera, Nathaly Alexandra Ramirez, Alejandro Gonzalez-Orozco, Isabella Caicedo, Paulo Cabrera

**Affiliations:** Fundación Cardioinfantil – Instituto de Cardiología, Bogotá, Colombia

**Keywords:** Pneumoperitoneum, Pneumatosis intestinalis, Portal venous gas, Gastrostomy, Case report

## Abstract

**Introduction:**

The gastrostomy is one of the most common procedures performed in general surgery. Although a simple procedure, it is not exempted from potential complications, specifically portal venous gas and intestinal pneumatosis being some of the ones with higher rates of mortality. The following case report presents a pneumoperitoneum due to extensive pneumatosis from esophageal, gastric, intestinal and portal gas. These rare complications were managed medically without undergoing emergency surgical intervention.

**Presentation of Case:**

A 19-year-old male patient, with previous history of cerebral palsy, chronic malnutrition and severe physical deconditioning, required a nutritional access. Due to co-existing pathologies, an open gastrostomy was chosen as the best intervention, which was performed without complications. On the tenth postoperative day, patient presents abdominal pain and diarrhea; laboratory results were within normal limits, and the abdominal computed tomography scan reported extensive pneumatosis compromising esophagus, stomach, small intestine, part of the colon, pneumoperitoneum and gas in the portal venous system. Medical management was carried out with an adequate recovery.

**Discussion:**

Intestinal pneumatosis and portal venous gas are rare and potentially lethal complications. Surgical intervention as well as severe malnutrition impairs carbohydrate digestion and promotes bacterial fermentation forming large volumes of gas and dissection of the intestinal mucosal wall, causing the intestinal pneumatosis evidenced in this case report.

**Conclusions:**

This case report presents a rare open gastrostomy complication, as well as a differential diagnosis to pneumoperitoneum. Additionally, the medical management poses a successful alternative to an emergency surgical intervention.

## Highlights

• Open gastrostomy lethal complications include intestinal pneumatosis and portal venous gas.
• Intestinal necrosis, disruption of mucosa, increased permeability of mucosa, and pulmonary disease, can cause complications.
• There are several theories describing pathophysiology of intestinal pneumatosis. one of them, secondary to surgery or trauma.
• Medical versus surgical management of the complications depend on the patient’s comorbidities and physician’s consideration.

## 1 Introduction

The gastrostomy is one of the most frequent procedures performed. Potentially, common gastric complications include: obstruction, tube displacement, bleeding and perforation. Meanwhile, portal venous gas and intestinal pneumatosis are rare complications. Pneumatosis as a complication was reported for the first time by the University of Michigan group in 1983 [1]. Radiologic imaging is the gold standard for diagnosis of pneumatosis and often associated with intestinal ischemia, obstruction and necrosis – all with high mortality rates [2]. In the literature, there is no established treatment for these cases. It remains a challenge whether a surgical or medical management is preferred. The following case report documents a patient who underwent an open gastrostomy and insidiously developed pneumoperitoneum with portal venous gas secondary to esophageal, stomach, and intestinal pneumatosis, reported in accordance with the SCARE criteria [3], at Fundación Cardioinfantil – Instituto de Cardiologia, in Bogota, Colombia.

## 2 Case presentation

A 19-year-old male patient, with body mass index (BMI) of 8.45 kg/m [2] and previous diagnosis of cerebral palsy, was admitted due to upper gastrointestinal bleeding Blatchford score of 10. Upon initial assessment, the patient required a blood transfusion due to hemoglobin level of 5.48 g/dl. The upper gastrointestinal endoscopy reported an esophageal ulcer Forrest IIC and esophagitis. Given the symptoms associated with chronic malnutrition and severe deconditioning, a gastrostomy was recommended. Initially an endoscopic gastrostomy was decided as the ideal approach, which was unsuccessful due to suboptimal translumination. Despite considering a new attempt to perform endoscopic gastrostomy at a later date, the patient´s nutritional and metabolic condition could worsen in case it failed a second time. Thus, an open gastrostomy was considered by the gastroenterology department to ensure an early start of the enteral nutritional route. The institutional anesthesiologist considered the patient’s high risk would be reduced once he was in adequate nutritional and metabolic so the surgery was performed without any initial complications. An upper gastrointestinal endoscopy on the third postoperative day revealed adequate positioning of the gastrostomy and enteral nutrition was initiated and well tolerated.

**Fig. 1:**
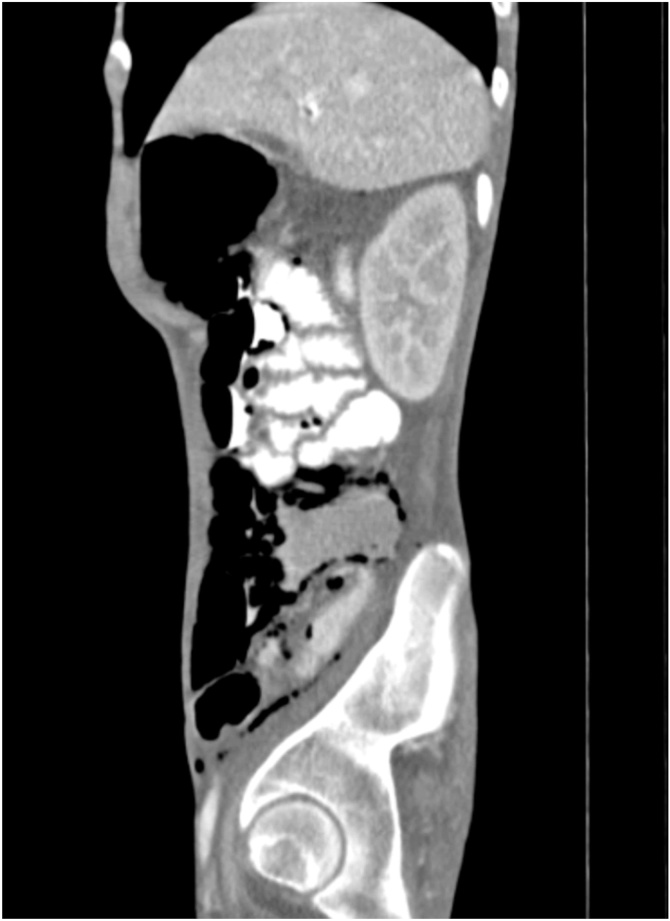
**Abdominal sagittal computed tomography image.** This abdominal reveals extensive pneumatosis, moderate pneumoperitoneum and gas in the venous portal system.

**Fig. 2:**
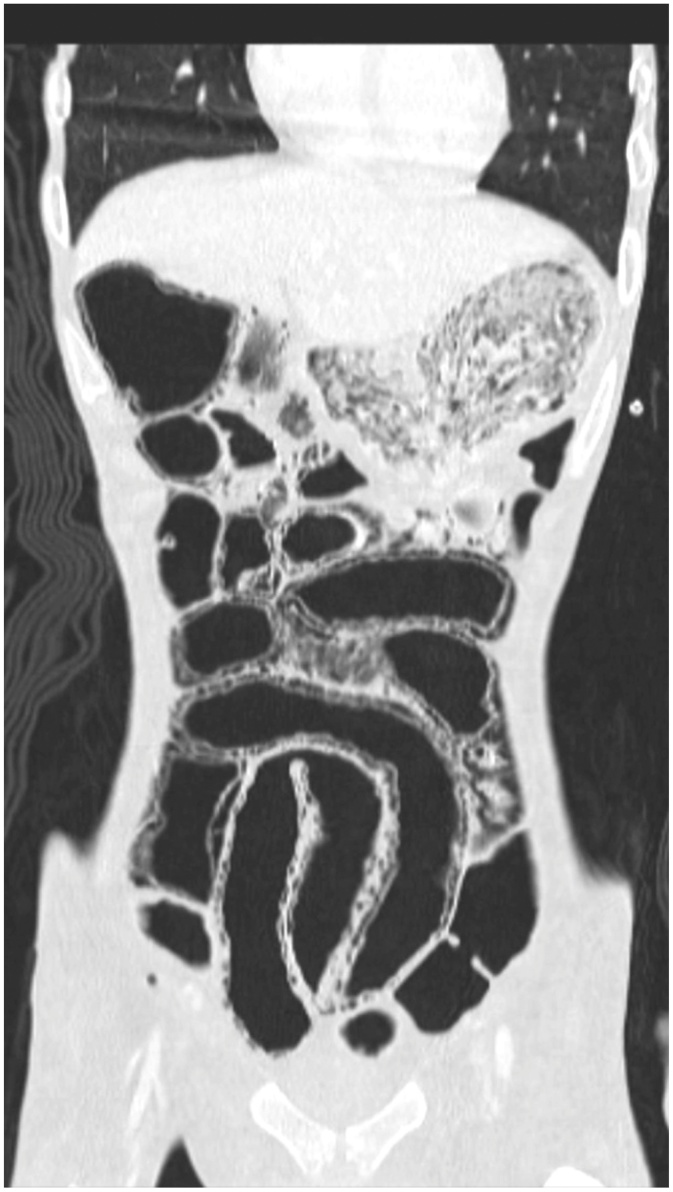
**Abdominal coronal computed tomography image.** This abdominal reveals extensive pneumatosis, moderate pneumoperitoneum and gas in the venous portal system.

Ten days after surgery, patient in-hospital presented diffuse abdominal pain and multiple diarrheic episodes, of insidious origin, referring it began two days after surgical procedure and gradually increased its intensity. Laboratory results were within normal limits, and the abdominal computed tomography (CT) scan revealed extensive pneumatosis from esophagus, stomach, small intestine and partial colon. Additionally, moderate pneumoperitoneum and gas in the venous portal system were also reported (Fig. 1, Fig. 2). The CT scan showed no evidence of an intra-abdominal collection or abscess that could otherwise explain the findings, as there was also no clinical or laboratory signs of systemic inflammatory response syndrome or infection. Medical management was initiated with intravenous fluids and nasogastric tube, while suspending the enteral nutrition. Patient showed improved outcome regarding symptomatology 24 h later. One month after the surgery, the patient was discharged in good conditions, with nutritional supplement via gastrostomy and integral rehabilitation.

## 3 Discussion

The following case reports a pneumoperitoneum, as a rare complication of open gastrostomy procedure, usually associated with a rupture of the digestive tract (90%) [4]. The ideal treatment is an urgent surgical approach, however a ruptured digestive tract is not always the cause of pneumoperitoneum; other causes can be thoracic, gynecologic, iatrogenic, or intestinal pneumatosis. In cases where a surgical approach is decided, the consequences need to be considered, as there is a high risk of requiring subsequent surgical interventions.

Intestinal pneumatosis and portal venous gas are rare and potentially lethal complications. Pneumatosis normally occurs in the small intestine [5] and rarely in the esophagus or stomach [6]. The extent of the pneumatosis in this case report has been reported on one previous occasion as a complication of a gastrostomy [1]. These complications can be caused by intestinal necrosis, disruption of the intestinal mucosa, increased permeability of the mucosa, and pulmonary disease.

The etiologies can be either benign or potentially lethal. Although their pathophysiology is not clear, several theories have been proposed [[7], [8], [9]]

Considering the patient in this case report was in an early postoperative state from the gastrostomy, theory four stemming from the surgical context (Table 1) could explain the complications. Additionally, the severe malnutrition can impair carbohydrate digestion and promote bacterial fermentation in the intestine, resulting in the formation of large volumes of gas and dissection of the intestinal mucosal wall [10]. Not to be excluded, the immunosuppression caused by the severe malnutrition state can also contribute to the extensive intestinal pneumatosis evidenced in this case report.

**Table 1:** Theories proposed by diferent authors explaining neumoperitoneum physiopathology.

**Theory 1** Imbalance between the luminal gas composition and pressure creates a supersaturated gas forming air bubbles in the vessels of the intestinal walls	**Theory 2** Proliferation of bacteria increments the lumen gas pressure and penetrates the mucosa barrier causing damage
**Theory 3** Secondary to pulmonary disease, an increase in alveolus pressure causes rupture and pneumomediastinum	**Theory 4** In surgery or trauma, the increase of abdominal pressure causes the intraluminal air to penetrate the intestine wall

Clinically, some patients are asymptomatic while others present severe symptoms of abdominal pain and diarrhea (53%) [10] followed by abdominal distension, nausea, vomit, and bloody or mucous stool. The CT scan is the diagnostic gold standard [11], and it is usually used again in patient follow-up.

The treatment protocol has varied in the last years from a medical approach to an early surgical intervention. Treatment is often not warranted in asymptomatic patients as they resolve spontaneously [7]. Symptomatic patients require a medical approach consisting in: intestinal decompression, parenteral nutrition, fluid and electrolyte repletion, and in defined cases antibiotics [12]. The surgical approach for these patients remains controversial, especially considering that a delay in the intervention can be harmful. Several algorithms have been published to aid in the decision process for the surgeon [[13], [14], [15]].

## 4 Conclusions

Pneumoperitoneum is a potentially rare complication of open gastrostomy, which can be treated medically as done in this case report. Nevertheless, potentially lethal conditions such as mesenteric ischemia, intestinal necrosis and intestinal obstruction have to be excluded first and treated accordingly, considering pneumoperitoneum as a differential diagnosis. A complete evaluation of the patient and a close follow-up using an established algorithm can prevent surgical interventions in benign cases of intestinal pneumatosis, as well as optimize the surgical intervention for those who require it early.

## Conflicts of interest

The authors declare they have no conflicts of interest.

## Sources of funding

This research did not receive any specific grant from funding agencies in the public, commercial, or not-for-profit sectors.

## Ethical approval

The Ethical and Research Committee of the Fundación Cardioinfantil – IC and the General Surgery Research Group at the Fundación Cardioinfantil – IC.

## Consent

Written consent was obtained from the patient for publication of this report. Any details identifying the individuals to the clinical history and images associated were eliminated as to remain anonymous.

## Author contribution

Perez Rivera CJ, González-Orozco A, and Caicedo I designed the report, analyzed the data, and wrote the paper. Ramirez NA, Cabrera P collected patient’s data and were the perioperative attending physicians.

## Registration of research studies

N/A.

## Guarantor

Perez Rivera Carlos Jose.

## Provenance and peer review

Not commissioned, externally peer-reviewed.
